# Turning over New Leaves

**Published:** 1888-06-30

**Authors:** 


### Turning Over New Leaves.

[Books for Review should be sent to The Editor, The Lodge, Porchester Square, W.]

### Paris in London.*

There is nothing new in these days but the undeniably antique! It is perhaps a testimony to the fundamental permanence of the characteristics of human nature, masked as they are by the disguises of changing fashions, that so many of our new novels avow themselves to be reproductions of old stories in modern dress. We have had "The New Republic," "The New Paul and Virginia," "The New Abelard," and "The New Antigone." The method has its advantages; it is really easy of invention, and gives the reader just enough information about the nature of the tale, without exposing the details of the plot too much. If, for example, we were writing a story which dealt with a young Englishman who went out to Australia and married a rich sheep-farmer's daughter, we should call it "The New Jason." On the other hand, if he jilted her for the sake of a "horsey" young lady at home he would pose as "The New Theseus," while if the antipodean damsel consoled herself with the love of a handsome youth whose faculties were sometimes dulled with wine she might monopolise the title-page as "The New Ariadne." If, however, we must be neo-classic in our nomenclature, in the name of Lemprière let us be correct. Now the title of "The New Judgment of Paris," is distinctly misleading. Although we have neither Homer nor the abovementioned classical dictionary at hand to refer to, we are distinctly of opinion that the Paris of the classic fable was a gentleman, and that in deciding, with the help of a bribe, on the claims of the rival goddesses to the award of beauty, he was dealing with a question which was, in the strictest sense at least, impersonal. Now Paul Lafargue's Paris is a young lady, Miss Ida Bannatyne—perhaps that is where the connexion comes in; Paris lived on the slopes of Mount Ida—and the question on which she gives judgment is the very personal one of which of three gentlemen she will accept as a husband. Putting aside the question of classical accuracy, however, the reader will approve of Miss Bannatyne's final choice, the much indebted young soldier, Eric Armstrong. She is cynical, worldly, and a trifle vulgar; he has good looks, a good position, a moral nature much on a par with her own, and an intellect decidedly inferior; he is the very mate for such a woman. The two other admirers, Ambrose Trevor, the artist, who speaks much truth in those rhapsodies in which he indulges on the slightest provocation, and John Sumner, the clever young engineer, are far too good for her. With such a heroine the book can hardly be a pleasant one; but it is undeniably clever. That is, it is the work of a clever man with a shrewd eye for surface truth and a turn for paradox and epigram. The writing is consistently good and vigorous, the descriptions of various scenes are apt and true; but the dialogue strains too visibly after smartness to be natural. There is no illusion; perhaps the author does not want it, and has no desire that his own wit should be too completely appropriated by his characters. Of these characters the so-called minor ones are decidedly the best and most interesting. We hope indeed that Dr. Harvey Bland, the blarneying, money-lending Irish physician, is not drawn from life, though the vigour with which he is sketched suggests personal acquaintance; and Sister Irene, the high-purposed but dogmatic nurse, who strives to rule like an autocrat in her hospital, and sets herself up as rival to the doctors and governors in place of being their helper, represents a type with which we are becoming daily more and more familiar. As we have said, the book is clever and amusing, well worth spending a few leisure hours in reading, though it may chance to leave the reader somewhat sadder for all its brightness. And when next "Paul Lafargue" takes up his pen to tell a story let him turn his eyes from the cross-lights that illuminate the cynic's domain, and going out into the highways and byways if need be—anywhere that the light of heaven, however obscured by London smoke, can reach straight down to—tell us what he sees there. The world is a less witty and a less worldly place than he depicts it, and eyes that pierce below the surface, as a scientist's should do, will see even in this life the germs of those qualities that justify our hopes of immortality.

* "The New Judgment of Paris." A novel. By Paul Lafargue. (London: Macmillan and Co.) Two volumes.

### Posological and Therapeutic Tables.*

Doctors have a liking for long and learned words, which is absolutely ineradicable. The number of new terms which is annually employed in the description of medical entities of various kinds, more particularly by senior students and those marvels of universal knowledge and wisdom, the first year's graduates, is almost past belief. Goldsmith's schoolmaster was a mere alphabetarian compared with a newly-fledged doctor. This little book, which has such a sesquipedalian name, will go into the waistcoat pocket; and its object is to tell nurses and medical students the doses of the various drugs in general use, and the effects which the drugs are ordinarily intended to produce. It is, in fact, a series of tables of the doses and uses of drugs. Every nurse should have either it, or a handy volume like it. It is quite reliable and, we believe, cheap; though the publishers, in spite of our repeated requests to the contrary, cannot be got to tell us the prices of their wares. The present is the third edition. The book has been a favourite with students and nurses for a dozen years.

* By A. Henry, M.D. Edinburgh: MacLachlan and Stewart.

### Magazines of the Month.

The *Universal Review* for this month shows no falling-off from the high standard of the first number, but indeed rather an improvement. The exquisite reproduction of Rossetti's beautiful picture "La Bella Mano" is in itself worth paying the price of the whole journal for, and the letterpress proves that grave subjects can be treated lucidly and thoughtfully, without an atom of dulness in the writing. Mr. Quilter makes the subject of the Salon as interesting as he did that of the Academy last month. Mr. Frank Hill and Mr. Grant Allen express with equal wit and earnestness political views that show considerable divergence, for the "Universal Review" belongs to no party, but opens its pages to all sides of great questions. Professor Freeman deals cleverly with Mr. Lowell's dictum that the first duty of a nation is to grow great men; and Mr. Wilkie Collins tells some quaint experiences in his "Reminiscences of a Story-Teller." Mr. Haweis's "tract for the times"—"The Parson, the Play, and the Ballet"—is a manly and honest utterance on a subject on which much nonsense has been talked. Mr. William Archer is on familiar ground in "A Sixteenth-Century Playhouse," and the other articles make up a number full of varied and interesting information.

The *National Review* contains a grave and thoughtful article on "The Creed of the Poor," by Mrs. Jeune. "The question is often asked," she says, "what is the secret of the patience of the poor, when we see men and women in prosperous positions in life well-nigh succumb to misfortunes insignificant in comparison?" The answer given is this: "The faith of the poor is, I am convinced, summed up in their strong belief in immortality, and to them an immortality of happiness and peace. A belief in the justice of God and the existence of Heaven is the epitome of the faith of the poor, and the hope born of that belief makes their lives on earth bearable. . . . . The Heaven they believe in will be theirs if in patience, honesty, and sobriety they live their life on earth. The poor neither care for nor understand the dogma of the Christian religion. The rock on which they build their faith is the teaching of Christ denuded of all doctrinal mysteries, and the pure lofty unselfishness of His character appeals to their weary and sorrowful souls." Indeed, the quiet grasp of the essentials of true religion by the poor—clung to the more firmly, it may be, because they have not time to "bother about" the subtle qualms of the intellectual agnostic and the elaborate ceremonies of the sensuously æsthetic—still bears witness to the truth of the Bible saying, that the facts of eternal life are hidden from the wise and prudent and revealed to babes. While speaking with full appreciation, however, of the noble qualities of the poor, Mrs. Jeune admits sadly that no teaching seems able to impress on them the folly and improvidence of early marriages.

IN *The Century*, Mr. George Keenan, dealing with the "Plains and Prisons of Western Siberia," describes the hospital at the prison at Tisunen thus: "The hospital wards, which numbered five or six, were larger and lighter than any of the cells that we had previously examined in the main building, but they were wholly unventilated, no disinfectants apparently were used, and the air was polluted to the last possible degree. It did not seem to me that a well man could live there a week without becoming infected with disease, and that a sick man should ever recover in that awful atmosphere was inconceivable. In each ward were twelve or fifteen small iron bedsteads, set with their heads to the walls round three sides of the room, and separated one from another by about five feet of space. Each bedstead was furnished with a thin mattress, consisting of a coarse grey bed-tick filled with straw, a single pillow, and either a grey blanket or a ragged quilt. Prisoners suffering from malignant typhus fever were isolated in a single ward; but, with this exception, no attempt apparently had been made to group the patients in classes according to the nature of their diseases. Women were separated from the men, and that was all. Never before in my life had I seen faces so white, haggard, and ghastly as those that lay on the grey pillows in these hospital cells. The patients, both men and women, seemed to be not only desperately sick, but hopeless and heart-broken."

*Work and Leisure* contains an article on "The Charge of Workhouses an Occupation for Gentlewomen," which should help to give practical effect to the growing conviction that the matron of a workhouse ought to be a lady. A woman of inferior education and up-bringing is very apt to prove the proverbial "beggar on horseback" when set over others who are practically her equals, though we must protest that we do not use the word gentlewoman in any limited sense, but take it according to Walter Besant's definition as meaning a woman with refined ideas and an educated mind.

WITH the summer a new "magazine for the people" has begun its career. *The Sun* is as bright as its title. Its supply of three serial stories may seem superabundant to critics, but probably its readers will find no fault therewith, while the articles on science and folk-lore contain much information in a pleasant and unpretentious form, and the religious papers show a high level of thought and feeling, untainted by any narrow sectarianism.

